# Supplementary material for: Leveraging host-genetics and gut microbiota to determine immunocompetence in pigs
Source: Anim Microbiome. 2021 Oct 24;3:74. doi: 10.1186/s42523-021-00138-9 (PMC8543910; doi:10.1186/s42523-021-00138-9)

Supplementary figure S1. Iris-plot representing the 20 most abundant genera. Each bar represents a sample, and bar colors represented the genera relative abundance.

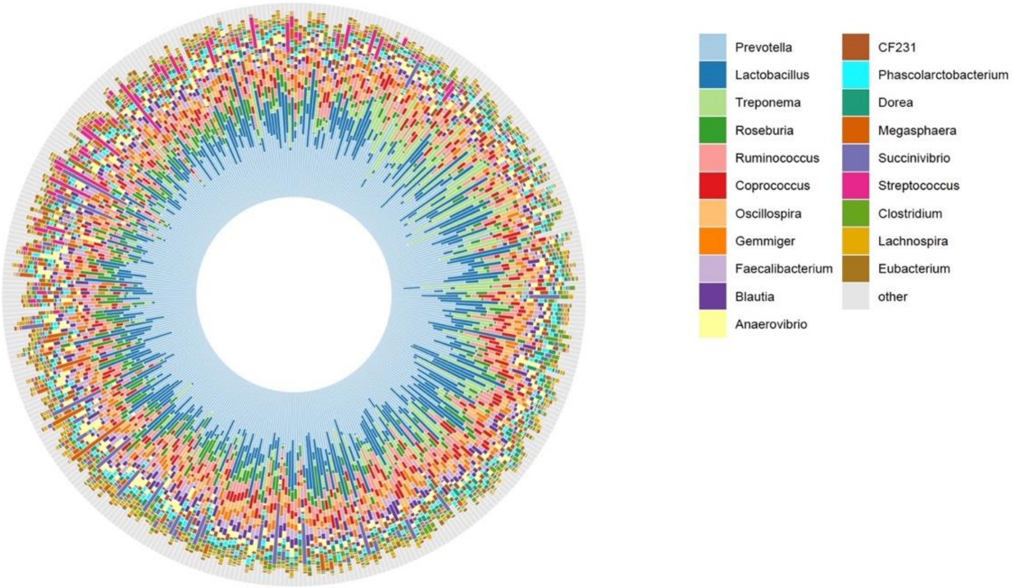

Supplement: Supplementary file 1 — Additional file 1. Figure 1. Iris-plot representing the 20 most abundant genera. Each bar represents a sample, and bar colors represented the genera relative abundance. [file 42523_2021_138_MOESM1_ESM.pdf]
