# Supplementary material for: Leveraging host-genetics and gut microbiota to determine immunocompetence in pigs
Source: Anim Microbiome. 2021 Oct 24;3:74. doi: 10.1186/s42523-021-00138-9 (PMC8543910; doi:10.1186/s42523-021-00138-9)
Supplement: Supplementary file 2 — Additional file 2. Table 1. Posterior estimates of h2 and m2 for the 21 health-related traits. [file 42523_2021_138_MOESM2_ESM.pdf]

Trait	Full name	h2	sd	P(h2>0.1)	m2	sd	P(m2>0.1)	ILh2G	SLh2G	ILh2M	SLh2M
CRP	C-reactive protein	0.142	0.046	0.900	0.180	0.059	0.942	0.073	0.254	0.068	0.291
EOS	eosinophils	0.186	0.061	0.962	0.207	0.063	0.986	0.082	0.316	0.094	0.331
γδ T-cells	gamma-delta T cells	0.147	0.067	0.819	0.152	0.053	0.851	0.063	0.201	0.055	0.258
GRANU_PHAGO_FITC	mean fluorescence in FITC among granulocytes	0.255	0.061	1.000	0.234	0.056	1.000	0.135	0.371	0.129	0.341
GRANU_PHAGO_%	percentage of phagocytic granulocytes	0.199	0.052	0.995	0.180	0.062	0.933	0.100	0.300	0.068	0.300
HP	Haptoglobin	0.138	0.040	0.853	0.276	0.082	0.996	0.066	0.210	0.123	0.435
IgA	immunoglobulins IgA	0.220	0.081	0.996	0.217	0.073	0.980	0.101	0.373	0.075	0.355
IgAsal	IgA saliva	0.202	0.051	0.997	0.210	0.073	0.975	0.116	0.309	0.081	0.351
IgG	immunoglobulins IgG	0.316	0.092	1.000	0.176	0.059	0.939	0.147	0.486	0.074	0.293
IgM	immunoglobulins IgM	0.359	0.087	1.000	0.158	0.046	0.909	0.193	0.535	0.069	0.246
LEU	total number of leukocytes	0.201	0.057	0.997	0.177	0.054	0.948	0.104	0.301	0.076	0.279
LYM	lymphocytes	0.194	0.055	0.972	0.214	0.063	0.985	0.091	0.294	0.094	0.331
LYM_PHAGO_FITC	mean fluorescence in FITC among lymphocites	0.242	0.054	1.000	0.260	0.053	1.000	0.145	0.332	0.155	0.362
LYM_PHAGO_%	pertcentage of phagocytic lymphocytes	0.207	0.051	0.991	0.238	0.066	0.998	0.111	0.298	0.109	0.365
MON_PHAGO_FITC	mean fluorescence in FITC among monocytes	0.200	0.050	0.998	0.196	0.052	0.994	0.109	0.292	0.100	0.294
MON_PHAGO_%	pertcentage of phagocytic monocytes	0.189	0.060	0.978	0.219	0.065	0.993	0.087	0.298	0.101	0.343
MONOCITOS_MM	monocytes	0.153	0.036	0.972	0.173	0.054	0.944	0.091	0.223	0.077	0.277
NO	Nitric Oxide	0.211	0.066	0.988	0.231	0.078	0.983	0.092	0.349	0.086	0.377
PHAGO_FITC	mean fluorescence in FITC among all phagocytic cells	0.256	0.064	1.000	0.220	0.055	0.999	0.134	0.381	0.113	0.322
PHAGO_%	percentage of total phagocytic cells	0.157	0.047	0.946	0.190	0.068	0.938	0.081	0.248	0.069	0.319
NEU	neutrophils	0.239	0.061	1.000	0.155	0.050	0.884	0.122	0.352	0.056	0.252
